# Impact of Primary Care Visit Frequency on Non-Urgent Emergency Department Visits in a Large Urban Medical Center

**DOI:** 10.1177/21501319261420566

**Published:** 2026-02-14

**Authors:** Akhil Chandekar, Olutola Akande, Christian Makar, Kyrillos Grace, Suha Abduselam, Omotoluwafe Balogun, Marisa Nwoke, Chika Okeke, Meron Gebreyes, Joni Ricks-Oddie, Soheil Sadaat, Bharath Chakravarthy, Candice Taylor Lucas

**Affiliations:** 1 Department of Emergency Medicine, School of Medicine, University of California, Irvine, CA, USA; 2 PRIME Leadership Education to Advance Doctoring - African, Black and Caribbean (PRIME LEAD-ABC), School of Medicine, University of California, Irvine, CA, USA; 3 Department of Medicine, UC San Diego School of Medicine, San Diego, CA, USA; 4 Department of Anesthesiology & Pain Medicine, University of Washington, Seattle, WA, USA; 5 Department of Emergency Medicine, University of California, Los Angeles, CA, USA; 6 Department of Medicine, Cedars-Sinai, Los Angeles, CA, USA; 7 Biostatics, Epidemiology and Research Design Unit, Institute for Clinical and Translational Sciences, University of California, Irvine, CA, USA; 8 Department of Statistics, Center for Statistical Consulting, University of California, Irvine, CA, USA; 9 Department of Pediatrics, School of Medicine, University of California, Irvine, CA, USA

**Keywords:** non-urgent emergency department visits, primary care visit frequency, emergency department overcrowding, patient navigation, care coordination, healthcare access barriers

## Abstract

**Purpose:**

To examine whether primary care visit frequency (PVF) is associated with non-urgent emergency department visits (NU-EDVs) at a large urban medical center, and to identify determinants of higher PVF among Emergency Department patients.

**Methods:**

We conducted a cross-sectional survey of adult ED patients (ESI ≥4 considered NU-EDV) at a Southern California Level I trauma center (September 2021-April 2024). Undergraduate research associates administered a 29-item instrument capturing demographics, utilization, barriers, trust in primary care, and comorbidities. Bivariate tests and multivariable logistic regression estimated adjusted odds ratios (aOR) for NU-EDV and for PVF ≥3 visits/year.

**Results:**

Among 964 patients with a primary care provider, 62.9% reported <3 PCP visits/year; 59.2% presented with NU-EDV. After adjustment, PVF ≥3/year was associated with lower odds of NU-EDV (aOR 0.64, 95% CI 0.48-0.87). Medicaid/Cal insurance was associated with higher odds of NU-EDV versus private insurance (aOR 1.53, 95% CI 1.12-2.08). Determinants of PVF ≥3/year included female sex (aOR 1.39, 95% CI 1.04-1.86), older age (aOR 1.01 per year, 95% CI 1.00-1.02), Black race (aOR 2.21, 95% CI 1.17-4.19), Medicaid/Cal coverage (aOR 1.70, 95% CI 1.22-2.37), more chronic conditions (aOR 1.53 per condition, 95% CI 1.35-1.71), and lower odds with PCP distrust (aOR 0.54, 95% CI 0.30-0.96).

**Conclusions:**

Greater primary care engagement is independently associated with fewer NU-EDVs; however, Medicaid beneficiaries remain at elevated risk for non-urgent ED use. Improving after-hours access, care coordination, and Medicaid-eligible unscheduled primary care may further reduce avoidable ED utilization.

## Introduction

Non-urgent emergency department (ED) visits (NU-EDVs) have become a significant public health issue both locally and globally. An estimated 13.7% to 27.1% of ED visits in the United States could have been treated at either a retail clinic or urgent care center.^1^ ED crowding not only causes logistical inconveniences but also significantly compromises patient outcomes and healthcare system efficiency by delaying care for true emergencies and straining healthcare resources.^2^ Analyzing NU-EDVs helps identify areas where public awareness and education about appropriate ED use can be improved. These visits may indicate problems with access to primary care or other outpatient services, highlighting areas for improvement in the broader healthcare system.

Primary care is a fundamental component of healthcare systems, serving as the first point of contact for patients and providing comprehensive, continuous care for a wide range of health issues. It broadly encompasses preventive care, health promotion, diagnosis and treatment of common illnesses, management of chronic conditions, and coordination of specialized care when needed. Extensive research has shown that primary care contributes to better health outcomes, more equitable health distribution, and lower healthcare costs.^3-5^ Improving access to primary care has been shown to decrease ED utilization and potentially reduce healthcare costs.^5-7^ Identifying the factors influencing non-emergent ED utilization is crucial for enhancing healthcare access, mitigating ED overcrowding, and ensuring patients receive appropriate care in the most suitable setting.

Meanwhile, there are factors that discourage utilizing primary care services and others that encourage ED utilization for non-urgent care. Some obstacles to primary care access include long wait-times for primary care appointments, unavailability of after-hours care, difficulty accessing primary care provider when ill, healthcare costs, lack of insurance coverage, lower socioeconomic status, perceived discrimination, and mistrust of primary care provider.^8-11^ Advantages associated with ED care include 24/7 accessibility, no upfront copay, and the availability of diagnostic tests and equipment.^12^ Therefore, this study sought to (1) explore the relationship between primary care visit frequency (PVF) and NU-EDVs within a large urban medical center, and (2) investigate the determinants of PVF among ED patients at a Southern California level 1 trauma medical center. Identifying these factors is imperative for developing targeted interventions that promote regular primary care visits, reduce non-urgent ED visits, and ultimately improve overall health outcomes.

## Methods

### Study Design and Setting

This study was reviewed and approved by the Institutional Review Board (IRB #4331) under an Exempt Self-Determination category, as no patient identifiers or protected health information were collected in the survey. All procedures were conducted in accordance with institutional guidelines and ethical standards. This study was conducted in the ED of an urban academic Southern California Level I Trauma Center. Participants were identified from the patient list by undergraduate research associates affiliated with the Emergency Medicine Research Associates Program (EMRAP). The screening process to identify potential participants was carried out daily from 8 AM to 12 AM (September 2021-April 2024) through Epic, the electronic health record software.

### Study Protocol

To be eligible for inclusion, patients had to be aged 18 years or older and have an Emergency Severity Index (ESI) of 4 or higher.^13^ The ESI is an ED triage algorithm that categorizes patients on a scale of 1 to 5 based on the severity of their condition, with 1 indicating the most severe and 5 indicating the least severe. Patients needing translation services, under 18 years of age, incarcerated individuals, or those under a 51/50 hold, were excluded from this study. An ESI ≥ 4 was defined as a non-urgent ED visit while 1 to 3 were issues requiring urgent medical attention.

### Data Collection

Upon identification of eligible patients, verbal consent was obtained before administering the survey. EMRAP associates verbally administered a 29-item survey using the Research Electronic Database Capture (REDCap) program, reading questions verbatim from a standardized script to ensure consistent delivery across participants. The Program in Medical Education Leadership Education to Advance Doctoring – African, Black and Caribbean (PRIME LEAD-ABC) Confronting Anti-Blackness with Research (CABR) Survey is a comprehensive questionnaire designed to assess healthcare-seeking behavior and perceptions among patients presenting to the ED of an urban academic medical center. The survey was developed in 2020 to 2021 by the study team in close collaboration with academic emergency physicians at an urban academic medical center during the COVID-19 pandemic, when EDs were experiencing unprecedented overcrowding and strain. Its content was informed by national surveys on ED utilization and access to care, as well as prior work on race, trust, and discrimination in health care, and was tailored to capture the specific needs and experiences of the local community, particularly African, Black, and Caribbean patients.^14^ The survey has not yet undergone formal psychometric validation as a standalone instrument; however, it was conceptually derived from validated instruments and refined with input from frontline ED clinicians to ensure clinical relevance and validity. It aims to understand patients’ reasons for visiting the ED and the perceived quality of care received, with the goal of improving emergency healthcare delivery. The survey covers various aspects, including demographic characteristics, reasons for ED visit, healthcare utilization patterns, perceptions of care, and experiences with healthcare providers. Data obtained from patients include demographic information such as age, gender identity, race, ethnicity, education level, and health insurance status. Additionally, patients provide details about their current health issue, reasons for choosing the ED over primary care, referral sources, existing medical conditions, language preferences for health information, past ED visits, experiences with healthcare during the COVID-19 pandemic, perceptions of care received, and attitudes towards healthcare disparities. The survey was either conducted verbally by the researcher or completed by the patient themselves.

### Statistical Analysis

All variables, including the number of primary care visits per year and number of chronic conditions was based on self-report in the survey. Chi-square tests were performed to identify statistically significant associations between yearly primary care provider visits and demographic variables (age, sex, race, education), clinical factors (comorbidities), and utilization-related variables such as, insurance type, patients’ perceived level of trust in their PCP, the presence of at least 1 barrier to accessing a PCP, reporting having at least 1 chronic disease, and whether the ED visit was preventable or non-preventable, as determined by the ESI assigned to patients on arrival at the ED.

An ESI score of 4 or 5 was classified as a preventable ED visit, while scores 1, 2, or 3 were classified as non-preventable visits. Chi-squared tests were also used to identify statistically significant associations between NU-EDV and demographic, clinical, and utilization-related variables mentioned above. Associations between identified statistically significant variables and the likelihood of NU-EDV were examined using multivariable regression models. We included certain non-significant variables in the model which, based on existing literature, have been shown to be associated with NU-EDV. Odds ratios (OR) with 95% confidence intervals (CI) were estimated from the logistic regression coefficients using maximum likelihood estimation.

The dependent variable was NU-EDV status (non-urgent vs. urgent), while independent variables included demographics (age, sex, race, education), clinical factors (comorbidities, number of PCP visits), and utilization-related factors (insurance, barrier to PCP and PCP trust). The results were interpreted to understand the factors associated with NU-EDV, providing insights into healthcare-seeking behavior patterns among adult patients presenting to the ED. Statistical significance was set at an alpha level of 0.05. Three PCP visits per year was selected as the cutoff based on national utilization patterns (≈321 visits per 100 persons annually)^15^ and empirical evidence that 3 visits per year significantly improves uptake of preventive care services.^16^

## Results

A total of 964 patients, all of whom reported having a PCP, were included in analyses (Table 1). Among them, 606 (62.86%) reported visiting their PCP less than 3 times per year while 358 (37.14%) reported visiting at least 3 times a year. Notably, a higher proportion (57.18%) of frequent visitors (PVF ≥ 3 visits) were females, whereas the proportion of females and males among those who reported visiting <3 times a year was about the same (50.9% vs 49.1%, respectively). On average, those who reported visiting at least 3 times a year were significantly older with a mean age of 48.7 years, compared to those who reported visiting less than 3 times a year, among whom the mean age was 41.2 years (*P* < .001). Approximately 42% were White, 32% Latinx, 15% Asian, 5.2% Black, 1.4% Hawaiian, 0.9% Native American, and 2.8% identified as Other race. Medicaid/Cal was most common among patients at 42.22%, next to private insurance (41.18%), Medicare (15.77%), and Tricare (0.83%). When asked about their level of trust in their PCP, 92.53% reported that they trust their PCP. Less than half of the patients (42.74%) reported facing at least 1 barrier to visiting their PCP. Such barriers included transportation, wait time for visit, limited appointment availability, cost, poor relationship with PCP, job responsibilities, and caring for loved ones. Approximately 63% reported having at least 1 chronic disease including high cholesterol, diabetes, asthma, kidney disease, autoimmune disease, cancer, and mental health disease. Upon arrival at the ED, 59.23% were classified as having a NU-EDV, indicated by an ESI score of 4 or 5.

**Table 1.** Bivariate Analysis of PVF Against Key Variables (Column Percentages Shown).

Variables	Category	< 3 Visits per year n (%)	≥3 visits per year n (%)	N (%)	*P*-value
N		606 (62.86)	358 (37.14)	964	
Sex	Female (%)	307 (50.91)	203 (57.18)	510 (53.24)	.060
	Male (%)	296 (49.09)	152 (42.82)	448 (46.76)	
Age	Mean (SD)	41.23 (17.05)	48.67 (18.47)	—	<.001
	18-44 (%)	355 (58.58)	146 (40.78)	501 (51.97)	
	45-64 (%)	156 (25.74)	122 (34.08)	278 (28.84)	
	65 (%)	95 (15.68)	90 (25.14)	185 (19.19)	
Ethnicity^a^	Hispanic	247 (42.66)	136 (39.08)	383 (41.32)	.284
	Non-Hispanic	332 (57.34)	212 (60.92)	544 (54.68)	
Race^b^	Black (%)	22 (3.63)	29 (8.10)	51 (5.29)	.009*
	Latine (%)	214 (35.31)	98 (27.37)	312 (32.37)	
	Asian or Asian American (%)	94 (15.51)	50 (13.97)	144 (14.94)	
	Native American or Alaskan Native (%)	5 (0.83)	4 (1.12)	9 (0.93)	
	Native Hawaiian and Pacific Islander (%)	7 (1.16)	6 (1.68)	13 (1.35)	
	White (%)	244 (40.26)	164 (45.81)	408 (42.32)	
	Other (%)	20 (3.3)	7 (1.96)	27 (2.8)	
Education	No HS diploma (%)	52 (8.58)	46 (12.85)	98 (10.17)	.039
	HS diploma (%)	154 (25.25)	94 (26.26)	248 (25.73)	
	Trade School / Associate’s degree (%)	56 (9.24)	35 (9.78)	91 (9.44)	
	Some college (%)	123 (20.3)	86 (24.02)	209 (21.68)	
	Bachelors (%)	153 (25.25)	65 (18.16)	218 (22.61)	
	Postgrad (%)	68 (11.22)	32 (8.94)	100 (10.37)	
Insurance	Medicaid/Cal (%)	239 (39.44)	168 (46.93)	407 (42.22)	.002*
	Medicare (%)	91 (15.02)	61 (17.04)	152 (15.77)	
	Private (%)	274 (45.21)	123 (34.36)	397 (41.18)	
	Tricare (%)	2 (0.33)	6 (1.68)	8 (0.83)	
Clear PCP instruction	Agree (%)	573 (94.71)	344 (96.63)	917 (95.42)	.169
	Disagree (%)	32 (5.29)	12 (3.37)	44 (4.58)	
PCP trust	Agree (%)	553 (91.25)	339 (94.69)	892 (92.53)	.050
	Disagree (%)	53 (8.75)	19 (5.31)	72 (7.47)	
Barrier to PCP access	Yes (%)	261 (43.07)	151 (42.18)	412 (42.74)	.787
	No (%)	345 (56.93)	207 (57.82)	552 (57.26)	
Chronic disease	Yes (%)	326 (53.80)	281 (78.49)	607 (62.97)	<.001
	No (%)	280 (46.20)	77 (21.51)	357 (37.03)	
Visit urgency	Urgent EDV (%)	221 (36.47)	172 (48.04)	393 (40.77)	<.001
	Non urgent EDV (%)	385 (63.53)	186 (51.96)	571 (59.23)	

a Hispanic/non-Hispanic was collected as an ethnicity variable, independent of race. Participants could identify as Hispanic and report any race or combination of races.

b Participants were allowed to check multiple racial categories.

* *P*-value was obtained using Fisher’s exact test.

Table 1 presents univariate analysis between annual PVF, participant demographics, and potential influential factors. Among the variables examined, sex, ethnicity, patient report of receiving clear instruction from their PCP, and report of facing a barrier to PCP access were not significantly associated with PVF (*P* > .05). Race was significantly associated with PVF (*P* = .009).

Commensurate with the racial distribution in the sample, White patients were the most represented group among patients who reported visiting their PCP less than 3 times (40.26%) or more than 3 times a year (45.81%). Whereas most people of other races reported visiting <3 times a year, a greater proportion (29 of 51) of Black patients who reported visiting their PCP more than 3 times a year. Across all levels of education, a higher proportion of patients reported visiting their PCP less than 3 times a year (*P* = .029). Insurance type was also associated with PVF (*P* = .002). Most patients with insurance (except for patients with Tricare) reported visiting their PCP <3 times a year. The patients’ report of trust in their PCP was marginally associated with PVF (*P* = .050). In both categories of reported visit frequency, most patients agreed to trusting their PCP. The patients’ report of having a chronic disease was significantly associated with PVF (*P* < .001). Among patients who reported visiting their PCP <3 times a year, 53.8% reported having a chronic disease, compared to 78.49% among those with 3 or more annual visits. The urgency of ED visit was also significantly associated with PVF (*P* < .001). A greater proportion of patients who reported visiting their PCP <3 times a year (63.53%) presented to the ED for a NU-EDV, compared to the proportion among those who reported visiting their PCP at least 3 times (51.96%).

Table 2 describes the bivariate analyses of factors associated with NU-EDV; PVF demonstrated the strongest association, with 67.4% of NU-EDV patients reporting fewer than 3 PCP visits per year compared to 56.2% of urgent visit patients (*P* < .001). Several other factors also showed statistically significant bivariate relationships with NU-EDV. Younger patients were more likely to present with non-urgent conditions (mean age 42.1 years vs. 46.7 years for urgent visits, *P* = .047), with 55.2% of 18-44 year olds having NU-EDV compared to 47.3% with urgent visits. Patients reporting barriers to PCP access were overrepresented among NU-EDV cases (45.4% vs. 38.9%, *P* = .047), as were those with Medicaid insurance (45.7% vs. 37.2%, *P* = .069) and self-reported chronic conditions (60.3% vs. 66.9%, *P* = .035), though chronically ill patients appeared to appropriately seek more urgent evaluation. No significant differences were observed by sex (*P* = 0.984), race/ethnicity (*P* = 0.284-0.857), education (*P* = 0.330), PCP trust (*P* = 0.681), or perceived clarity of PCP instructions (*P* = 0.766).

**Table 2.** Bivariate Analysis of NU-EDV Against Key Variables (Column Percentages Shown).

Variables		Non-urgentn (%)	Urgentn (%)	N (%)	*P*-value
	Category				
N		571 (59.23)	393 (59.23)	964	
Sex	Female (%)	302 (53.26)	208 (53.20)	510 (53.24)	.984
	Male (%)	265 (46.74)	183 (46.80)	448 (46.76)	
Age	Mean (SD)	42.10 (17.49)	46.74 (18.27)	—	<.047
	18-44 (%)	315 (55.17)	186 (47.33)	501(51.97)	
	45-64 (%)	157 (25.50)	121 (30.79)	278 (28.84)	
	65+ (%)	99 (17.34)	86 (21.88)	185 (19.19)	
Ethnicity^a^	Hispanic	247 (42.66)	136 (39.08)	383(41.32)	.284
	Non-Hispanic	332 (57.34)	212 (60.92)	544 (54.68)	
Race^b^	Black (%)	32 (5.60)	19 (4.83)	51 (5.29)	.857*
	Latine (%)	189 (33.10)	123 (31.30)	312 (32.37)	
	Asian or Asian American (%)	88 (15.41)	56 (14.25)	144 (14.94)	
	Native American or Alaskan Native (%)	6 (1.05)	3 (0.76)	9 (0.93)	
	Native Hawaiian and Other Pacific Islander (%)	9 (1.58)	4 (1.02)	13 (1.35)	
	White (%)	231 (40.46)	177 (45.04)	408 (42.32)	
	Other (%)	16 (2.80)	11 (2.80)	27 (2.8)	
Education	No HS diploma (%)	59 (10.33)	39 (9.92)	98 (10.17)	.330
	HS diploma (%)	137 (23.99)	111 (28.24)	248 (25.73)	
	Trade school/associate’s degree (%)	59 (10.33)	32 (8.14)	91 (9.44)	
	Some college (%)	119 (20.84)	90 (22.90)	209 (21.68)	
	Bachelors (%)	140 (24.52)	78 (19.85)	218 (22.61)	
	Postgrad (%)	57 (9.98)	43 (10.94)	100 (10.37)	
Insurance	Medicaid/Cal (%)	261 (45.71)	146 (37.15)	407 (42.22)	.069*
	Medicare (%)	85 (14.89)	67 (17.05)	152 (15.77)	
	Private (%)	221 (38.70)	176 (44.78)	397 (41.18)	
	Tricare (%)	4 (0.70)	4 (1.02)	8 (0.83)	
Clear PCP instruction	Agree (%)	542 (95.25)	375 (95.66)	917 (95.42)	.766
	Disagree (%)	27 (4.75)	17 (4.34)	44 (4.58)	
PCP trust	Agree (%)	530 (92.82)	362 (92.11)	892 (92.53)	.681
	Disagree (%)	41 (7.18)	31 (7.89)	72 (7.47)	
Barrier to PCP access	Yes (%)	259 (45.36)	153 (38.93)	412 (42.74)	.047
	No (%)	312 (54.64)	240 (61.07)	552 (57.26)	
Chronic disease	Yes (%)	344 (60.25)	263 (66.92)	607 (62.97)	.035
	No (%)	227 (39.75)	130 (33.08)	357 (37.03)	
PCP visit frequency (PVF)	<3 Visits per year	385 (67.43)	221 (56.23)		<.001
	≥3 visits per year	186 (32.57)	172 (43.77)		

a Hispanic/non-Hispanic was collected as an ethnicity variable, independent of race. Participants could identify as Hispanic and report any race or combination of races.

b Participants were allowed to check multiple racial categories.

* *P*-value was obtained using Fisher’s exact test.

The Logistic model is adjusted for variables that were significantly associated with NU-EDV, Table 3 presents the result of multivariable logistic regression analysis estimating the odds of a NU-EDV. We calculated adjusted odds ratios (aOR) for this association and controlled the following covariates: sex, age, race, health insurance type, and chronic disease. We found that patients who reported visiting their PCP at least 3 times a year were 36% less likely to present to the ED for a non-urgent reason compared to those who reported visiting less than 3 times a year (aOR: 0.64, 95% CI: 0.48-0.87). Compared to patients with private insurance, Medicaid/Cal beneficiaries were 53% more likely to present to the ED for a non-urgent reason (aOR; 1.53, 95% CI: 1.12-2.08). Although the association between reporting having a chronic disease and NU-EDV was not statistically significant in the adjusted model, the association was statistically significant in the unadjusted model with 14% decreased odds of a NU-EDV among patients with a reported chronic disease (OR: 0.86, 95% CI: 0.78-0.95).

**Table 3.** Multivariable Regression Analysis of the Association Between PCP Visit Frequency (PVF) and Non-urgent Emergency Department Visits (NU-EDV).

	Unadjusted odds ratios			Adjusted odds ratios		
Category	OR	95% CI	*P*-value	OR	95% CI	*P*-value
PVF						
<3/year (Ref.)				—		—
≥3/year	**0.62**	**0.48-0.81**	**<.001**	**0.64**	**0.48-0.87**	**.004**
Sex						
Male (Ref.)	—	—	—	—	—	—
Female	1.00	0.77-1.30	.984	1.00	0.78-1.34	.990
Age	**0.99**	**0.98-0.99**	**<.001**	**0.99**	**0.98-1.00**	**.043**
Race						
White (Ref.)	—	—	—	—	—	—
Black	1.29	0.07-2.35	.405	1.57	0.84-2.96	.155
Latino	1.18	0.87-1.59	.286	0.95	0.68-1.33	.781
Asian	1.20	0.82-1.77	.348	1.16	0.78-1.75	.462
Native American	1.53	0.38-6.21	.550	1.49	0.35-6.30	.588
Hawaiian	1.72	0.52-5.69	.371	1.69	0.48-5.90	.413
Other	1.11	0.50-2.46	.789	1.04	0.45-2.43	.925
Health insurance						
Private (Ref.)	—	—	—	—	—	—
Medicaid/Cal	**1.42**	**1.07-1.90**	**.015**	**1.53**	**1.12-2.08**	**.008**
Medicare	1.01	0.69-1.47	.957	1.29	0.85-1.96	.234
Tricare	0.80	0.20-3.23	.750	0.93	0.18-4.91	.935
Chronic disease index^a^	0.86	0.78 - 0.95	**.02**	0.92	0.82-1.02	.136
Barrier to PCP access	1.30	1.00-1.69	**.048**	1.30	0.98-1.73	.064
Trust PCP						
Agree (Ref.)						
Disagree	0.90	0.556-1.673	.681	0.76	0.46-1.28	.304

Significant values (*P* < .05) are bolded.

a Calculated as the total number of self-reported chronic conditions selected from the survey list, treated as a simple count rather than a validated comorbidity score.

To assess which factors may influence the frequency of PCP visits, a multivariable logistic regression analysis estimating the odds of visiting a PCP ≥ 3 times a year was conducted (Table 4). Independent variables included sex, age, race, insurance type, presence of chronic disease, and level of trust in the PCP, as these factors have previously been associated with differences in primary care utilization. An adjusted regression model revealed that females were more likely than males to report visiting their PCP, with a 39% increase in odds (aOR 1.39, 95% CI 1.04-1.86). Each additional year of age was associated with a 1% increase in the odds of visiting a PCP ≥ 3 times a year (aOR 1.01, 95% CI 1.00-1.02). Compared to White patients, Black patients were more likely to report visiting their PCP ≥3 times a year with a 121% increase in the odds of visiting a PCP (aOR 2.21, 95% CI 1.17-4.91). In contrast, patients who identified as Latine were 35% less likely to report visiting a PCP ≥3 times a year (aOR 0.65, 95% CI 0.46-0.94).

**Table 4.** Binary Logistic Regression Model Predicting the Odds of Visiting a PCP ≥3 Times a Year.

	Unadjusted odds ratios			Adjusted odds ratios		
Category	OR	95% CI	*P*-value	OR	95% CI	*P*-value
Sex						
Male (Ref.)	1	—	—	1	—	—
Female	1.29	0.99-1.68	.060	**1.39**	**1.04-1.86**	**.028**
Age	**1.02**	**1.02-1.03**	**<.001**	**1.01**	**1.00 - 1.02**	**.003**
Race						
White (Ref.)	1	—	—	—	—	—
Black	**1.96**	**1.09-3.53**	**.025**	**2.21**	**1.17-4.19**	**.015**
Latino	**0.68**	**0.50-0.93**	**.015**	**0.65**	**0.46-.94**	**.021**
Asian	0.79	0.53-1.18	.247	0.92	0.60-1.41	.708
Native American	1.19	0.31-4.50	.797	0.81	0.20-3.29	.763
Hawaiian	1.28	0.42-3.86	.67	1.22	0.35-4.26	.757
Other	0.521	0.22-1.26	.148	0.74	0.29-1.86	.516
Health insurance						
Private (Ref.)	—	—	—	—	—	—
Medicaid/Cal	**1.57**	**1.17-2.09**	**.002**	**1.70**	**1.22-2.37**	**.002**
Medicare	**1.49**	**1.01-2.20**	**.043**	1.00	0.64-1.58	.99
Tricare	**6.68**	**1.33-33.58**	**.021**	4.02	0.64-25.37	.139
Chronic disease index^a^	1.58	1.42 - 1.75	<.01	1.53	1.35-1.71	<.01
Trust PCP						
Agree (Ref.)	—	—	—	—	—	—
Disagree	**0.58**	**0.34-1.01**	**.052**	**0.54**	**0.30-0.96**	**.037**

Significant values (*P* < .05) are bolded.

a Calculated as the total number of self-reported chronic conditions selected from the survey list, treated as a simple count rather than a validated comorbidity score.

Considering health insurance type, Medicaid/Cal beneficiaries were more likely to report visiting their PCP with a 70% increase in odds relative to individuals with private insurance (aOR 1.70, 95% CI 1.22-2.37). Medicare or Tricare were not significantly associated with PCP visits in the adjusted model. However, unadjusted for other factors, Medicare beneficiaries were 49% more likely to report visiting their PCP (OR 1.49, 95% CI 1.01-2.20), and patients with Tricare had 568% increased odds of reporting visiting their PCP (OR 6.68, 95% CI 1.33-33.58), although only 8 Tricare patients were represented in the sample. For each additional reported chronic disease a patient had, there was a 53% increase in the odds of reporting visiting their PCP at least 3 times a year (aOR 1.53, 95% CI 1.35-1.71). Distrust in one’s PCP was associated with the 46% decreased odds of reporting visiting a PCP ≥3 times a year (aOR 0.54, 95% CI 0.30-0.96).

## Discussion

This study explores the complex association between primary care visit frequency and non-urgent ED utilization. The main outcome of this study showed that patients who reported visiting their PCP at least 3 times per year had significantly lower odds of presenting to the ED for a non-urgent condition. In interpreting this finding, it is important to note that primary care visit frequency was derived from a specific survey item asking, “How often do you visit your primary care physician in a year?” with fixed response options (0, 1, 2, 3, or 4 or more visits), highlighting that this measure reflects patient self-reported utilization in a typical year. This finding supports prior research showing that increased primary care utilization reduces NU-EDV by improving chronic disease management, preventive care, and timely interventions.^17^ As for chronic disease and its role in ED utilization, our study found that, in the unadjusted model, patients with reported chronic diseases showed a 14% decreased odds of non-urgent ED visits; however, this association was not statistically significant in the adjusted model. This discrepancy suggests that factors such as age, socioeconomic status, and access to primary care, which were controlled for in the adjusted analysis, may confound the relationship between reported chronic disease status and ED utilization. This observation was also shown in a study analyzing ED visits for preventable conditions, which found that while various socioeconomic variables were significantly associated with ED utilization in univariable analyses, only specific factors like education level and private car ownership remained significant in multivariable models, indicating that certain demographics and socioeconomic factors can influence associations observed in unadjusted analyses.^18^ While our study did not directly assess broader, health system-level interventions, such interventions might help to address persistent gaps in access, particularly for Medicaid populations. Evidence-based strategies such as telehealth, expanded after-hours urgent care, hospital-at-home models, and primary care workforce expansion, are showing promise in reducing unnecessary ED use, but must be coordinated and equitably available across patient populations.^19-22^

Within this cohort, race was significantly associated with self-reported primary care utilization, with Black patients more likely than White patients to report visiting their PCP ≥3 times per year and Latine patients less likely to do so. These patterns may reflect underlying differences in access, continuity of care, or trust, and are likely more generalizable to Southern California where Latine patients comprise a larger proportion of the population, though less representative for Black patients given their lower regional prevalence. Future studies should investigate these structural and interpersonal factors driving race-based differences in primary care engagement to inform targeted strategies for reducing non-urgent ED utilization. Prior research suggests that barriers such as limited appointment availability and lack of extender care options have been identified as contributors to decreased primary care engagement.^23^ For low-income populations, this effect is compounded by transportation barriers and insufficient health system support, deterring timely appointment attendance and increasing missed visits. With the expansion of digital health tools, recent studies suggest that telemedicine and virtual hotlines may help bridge this gap. A comprehensive review found that integration of telehealth resulted in a significant reduction in ED visits, especially for chronic disease management and acute, low-acuity complaints.^19^ Other large-scale retrospective analyses show telemedicine triage for low-risk conditions leads to low follow-up rates in emergency settings, demonstrating cost-effectiveness and reduced crowding without compromising care quality.^20,21^

Extender care options, including after hour urgent care clinics or after hour primary care clinics offer evidence-based solutions; studies demonstrate that when clinics extend hours and facilitate same-day or walk-in access, patients are less likely to use the ED for non-urgent needs. Data shows that urgent care clinics have been associated with a 17% reduction in ED visits in certain regions in the United States, saving an estimated $3.3 billion annually by cutting non-urgent ED utilization.^22^ Nevertheless, for these reductions to be effective, urgent care centers must have consistent standards and capabilities (suturing, basic medications, diagnostics) so that they are a true alternative and not merely a referral step. Studies have shown that after-hours primary care incentives were associated with a reduction of 1.26 less-urgent ED visits per 1000 patients per month.^24^ After-hours primary care access is critical because many patients face competing responsibilities such as work schedules, childcare, or other obligations that make it difficult to attend appointments during traditional weekday hours of 9 am to 5 pm. For these individuals, limited availability outside standard business hours potentially means that urgent health needs go unaddressed, leading to reliance on EDs for non-urgent issues.

Besides extender care clinics and telemedicine options, patient navigation and Hospital-at-Home (HaH) programs may also serve as methods to engage with patients outside of the ED. Pilot patient navigation programs have been shown to significantly improve PCP follow-up attendance by 52% and decrease repeat ED visits by 32%.^25^ In our cohort, distrust in one’s primary care physician was associated with decreased odds of frequent PCP visits, suggesting that perceived lack of trustworthiness may undermine longitudinal care relationships in this ED population. Strengthening patient-provider trust through culturally responsive communication and continuity may represent a modifiable lever to enhance primary care utilization and address disparities in ED overuse. Given that the majority of patients in our study reported trusting their PCP, yet 61.9% reported visiting their PCP less than 3 times a year, patient navigation initiatives may help bridge the gap between PCP availability and patient engagement, ultimately reducing the burden on EDs. HaH programs have expanded rapidly since 2020 and now demonstrate robust evidence for reduced ED visits, improved patient-centered outcomes, lower 30-day readmissions, and significant system cost-savings.^26^ A 2023 analysis revealed a 7% readmission rate within 30 days for HaH, compared to 23% in traditional inpatient care, with lower rates of complications and improved patient experience.^27^ HaH models are especially relevant for Medicaid enrollees and high-risk populations, who otherwise face access and transportation barriers.

Despite the benefits of primary care engagement, our study found that Medicaid beneficiaries had 53% greater odds of using the ED for non-urgent conditions irrespective of self-reported PCP visit frequency. This paradox is also shown in previous studies, where Medicaid patients, although insured, were amongst the highest number of patients to report visiting the ED, citing long wait times and appointment availability issues.^18^ This suggests that while more primary care engagement reduces overall non-urgent ED reliance, it does not fully eliminate unnecessary visits, nor does it significantly shift the balance between urgent and non-urgent cases. This paradigm was also explored by Acquadro-Pacera et al^28^ who analyzed ED utilization patterns amongst migrant and non-migrant populations and found that higher primary care visit frequency did not necessarily correlate with lower ED use. Their study suggested that even when patients engage regularly with primary care, systemic barriers such as long wait times and limited appointment availability contribute to persistent ED reliance. This aligns with findings that indicate higher PCP visits may not always reflect discretionary use but rather signify unmet healthcare needs, particularly among vulnerable populations. Additionally, differences in patient demographics, healthcare system structures, and accessibility of outpatient services may explain this discrepancy.

Increasing primary care access is insufficient to reduce non-urgent ED visits without addressing broader systemic barriers and care coordination challenges. Integrated models, like those adopted by Kaiser Permanente, demonstrate that embedding in-network urgent care, telehealth, and after-hours access within the health system can lower downstream ED visits, as opposed to patchwork approaches with limited reimbursement and siloed services.^29^ Wu et al^30^ reported that gaps in Medicaid urgent care reimbursement often forced patients to choose between delayed primary care access or ED use, reinforcing disparities. This 2024 evidence roundup further emphasized that Medicaid policy must focus on patient-centered navigation, removing insurance-linked coverage silos, and ensuring available unscheduled care options address social determinants and clinical needs.^30^

Lastly, our study highlights an opportunity for health systems to save money by investing in the future of same day appointments and unscheduled primary care visits. Increased ED utilization continues to impact the health care system financially with 144.8 million visits in 2017 alone generating $76.3 billion in costs.^31^ Many of these visits may represent non-urgent conditions that could be managed at a lower cost. There seems to be potential for cost savings if patients are effectively redirected to outpatient unscheduled care. Evidence from the Veterans Health Administration demonstrates that primary care engagement directly reduces expenditures: the first primary care visit was associated with an average cost reduction of $3976 per patient, with an estimated $2.5 billion saved between 2016 and 2019 among high-risk patients.^7^ Yet, our findings reveal that Medicaid beneficiaries continue to rely on the ED at disproportionately high rates despite frequent PCP visits. This fact emphasizes the recognized negative impact of poverty on health outcomes, and potentially highlights systemic barriers preventing access to care, such as limited after-hours availability and long appointment wait times.^8-10^ Addressing this gap will require both expanding access to timely outpatient care and strengthening the workforce pipeline for primary care. Medical schools and health systems should expand accelerated primary care programs, enhance tuition-free and scholarship initiatives, and invest in strategies that make primary care a more sustainable and attractive career path.^32^ Such reforms would not only alleviate downstream ED overutilization but also promote a more cost-effective and equitable healthcare system.

### Limitations and Future Directions

Our study has several limitations. First, the reliance on self-reported PCP visit frequency introduces potential recall bias. Additionally, our cross-sectional study design prevents us from establishing causality between primary care engagement and non-urgent ED visits. Longitudinal studies are needed to determine the directionality of these associations. Another limitation is the underrepresentation of certain racial and ethnic groups. While our study included a diverse sample, Black patients comprised only 5.29% of our population. Because the study sample may not fully represent broader populations, the external generalizability of these findings is limited. Given the documented disparities in healthcare access and utilization, future research should aim to ensure broader representation to better capture the full spectrum of factors influencing primary care engagement and ED use. Additionally, our measurement of patient trust in their PCP was limited to a single survey item (“I trust my primary care doctor,” with response options ranging from Strongly Agree to Strongly Disagree). Trust in a provider is a multifaceted construct that cannot be fully captured by 1 question and would benefit from a more comprehensive assessment using multiple validated items. Moreover, patients may have been reluctant to disclose negative perceptions of their relationship with their primary care provider due to the sensitive nature of this topic. However, the potential impact of social desirability bias was mitigated by the fact that questionnaires were administered by pre-health volunteers rather than physicians, medical students, or nursing staff.

A further limitation is the ED study setting itself, where patients may experience fatigue, acute discomfort, divided attention from clinical care, and environmental stressors that could affect concentration, response quality, and willingness to disclose sensitive information about healthcare experiences. Future studies could address this by administering a similar survey instrument in primary care offices as a control comparison group to evaluate how study setting influences reporting of PCP engagement and ED utilization patterns.

Lastly, although we adjusted for multiple demographic and socioeconomic factors, other variables including health literacy and provider availability were not included in this study, further study is needed to evaluate how these factors may impact the relationship between PCP engagement and ED utilization. Future research should also be done in a longitudinal manner with purposive sampling and mixed methodologies to increase representation of ethnic and racial minority groups. This could provide greater understanding of qualitative factors associated with the relationship between PCP engagement and ED utilization. Further studies should also explore the effectiveness of interventions such as patient navigation programs, after-hours care expansion, and Medicaid policy changes in reducing NU-EDVs.

## Conclusion

This study contributes new evidence on patterns of primary care visit frequency and non-urgent ED utilization, demonstrating opportunities to enhance continuity and efficiency of care. Patients with higher PCP visit frequency were significantly less likely to present to the ED for a non-urgent condition. However, Medicaid beneficiaries had disproportionately high non-urgent ED use despite frequent PCP visits, indicating systemic barriers. Additionally, improving care coordination and after-hours urgent care and primary care access may further reduce unnecessary ED reliance. Future studies should evaluate targeted interventions to improve healthcare accessibility and reduce unnecessary ED reliance.

## Supplemental Material

sj-pdf-1-jpc-10.1177_21501319261420566 – Supplemental material for Impact of Primary Care Visit Frequency on Non-Urgent Emergency Department Visits in a Large Urban Medical Center Supplemental material, sj-pdf-1-jpc-10.1177_21501319261420566 for Impact of Primary Care Visit Frequency on Non-Urgent Emergency Department Visits in a Large Urban Medical Center by Akhil Chandekar, Olutola Akande, Christian Makar, Kyrillos Grace, Suha Abduselam, Omotoluwafe Balogun, Marisa Nwoke, Chika Okeke, Meron Gebreyes, Joni Ricks-Oddie, Soheil Sadaat, Bharath Chakravarthy and Candice Taylor Lucas in Journal of Primary Care & Community Health
