# Supplementary material for: Impact of Primary Care Visit Frequency on Non-Urgent Emergency Department Visits in a Large Urban Medical Center
Source: J Prim Care Community Health. 2026 Feb 14;17:21501319261420566. doi: 10.1177/21501319261420566 (PMC12906620; doi:10.1177/21501319261420566)
Supplement: sj-pdf-1-jpc-10.1177_21501319261420566 – Supplemental material for Impact of Primary Care Visit Frequency on Non-Urgent Emergency Department Visits in a Large Urban Medical Center [file sj-pdf-1-jpc-10.1177_21501319261420566.pdf]

# LEAD-ABC CABR Survey

Record ID ______________________

Who is filling out the questionnaire today?
◯ Patient
◯ Research Associate

Today's Date ______________________

**Information Disclosure:**

**You are being asked to participate in a research study that is being conducted at the UC Irvine School of Medicine in conjunction with the Confronting Anti-Blackness Through Research (CABR) Team. This study aims to understand health care provided by the emergency department and the reasons for visiting the ED. Responses will be used to improve the quality of care provided by the Emergency Department.**

**Your participation involves completing a survey which will take approximately 10-15 minutes. Participation in this study is voluntary. You can choose to participate, not participate, or choose not to answer certain questions without any negative consequences.**

**All answers from this survey are confidential and will be kept in a secure database. This data will be used in future research studies, but any identifiers will only be known to the research team.**

Consent
◯ I am at least 18 years old and I provide consent
◯ I do not consent.

**Disclaimer:**

**PLEASE READ. Your responses to the following survey questions will not be shared with your provider today or your primary care provider if you have one. Therefore, please be sure to tell the medical professional that you see today all necessary information regarding your visit. Thank you.**

1) Name ______________________

2) Date of Birth ______________________

3) Gender identity
◯ Male
◯ Female
◯ Transfemale/transwoman
◯ Transmale/transman
◯ Gender queer/gender nonconforrming
◯ Different identity

| | |
|---|---|
| 4) Race | ☐ Black, Caribbean<br>☐ White<br>☐ Asian<br>☐ American Indian<br>☐ Latino or Spanish Origin<br>☐ Native Hawaiian or Other Pacific Islander<br>☐ Other: please list |
| Other race | __________________________________ |
| 5) Ethnicity | ◯ Hispanic<br>◯ Non-Hispanic |
| 6) Are there any languages other than English that were spoken in your household while growing up? | __________________________________ |
| 7) Other than the US, what countries do you identify with? | __________________________________ |
| 8) Highest level of education: | ◯ No schooling completed<br>◯ Between preschool and 8th grade<br>◯ Some high school, no diploma<br>◯ High school graduate, diploma or the equivalent (for example: GED)<br>◯ Some college credit, no degree<br>◯ Trade/technical/vocational training<br>◯ Associate degree<br>◯ Bachelor's degree (e.g. BA, BS)<br>◯ Master's degree (e.g. MBA, MPH, etc.)<br>◯ Professional degree (e.g. MD/DO, DDS, PharmD, JD, etc.)<br>◯ Doctorate degree (Ph.D.) |
| 9) Do you have health insurance? | ◯ Yes<br>◯ No |
| If yes, please specify: | ◯ Medicaid/Medi-cal<br>◯ Medicare<br>◯ Private Provider<br>◯ TRICARE |
| 10) What health issue brings you into the Emergency Room today? | __________________________________ |

11) What brought you into the emergency room today instead of seeking care from a primary care physician/provider? (Select all that apply)

- ☐ My doctor is closed right now
- ☐ My regular doctor told me to come here
- ☐ The emergency department costs me less money than my usual source of care to resolve my health issue
- ☐ The emergency department takes less time than my usual source of care to resolve my health issue
- ☐ The wait time in the emergency department is shorter than the wait time at my usual source of care
- ☐ I prefer not to schedule an appointment
- ☐ The emergency department is more convenient than my usual source of care
- ☐ I think I am having a medical emergency, and I felt going to the hospital would have more testing available.
- ☐ I had nowhere else to go
- ☐ ER is closest provider
- ☐ This is my usual source of care
- ☐ Other: (please explain)

Other: (please explain) __________________________________

12) Did a medical professional tell you to come or a family member?

- ◯ Doctor/Nurse
- ◯ Family/Friends
- ◯ No one told me to come in today

Who and what's your relation to this person? __________________________________

13) Do you have any of the following medical conditions? (please select all that apply)

- ☐ High Blood Pressure
- ☐ High Cholesterol
- ☐ Diabetes
- ☐ Asthma
- ☐ Kidney disease - problems with kidneys
- ☐ Dialysis
- ☐ Smoking
- ☐ Autoimmune disease - Lupus, HIV, rheumatoid arthritis
- ☐ Cancer
- ☐ Mental Health Disease - Depression, Anxiety, Bipolar disorder, Schizophrenia
- ☐ None
- ☐ Other: please list

Other: please list __________________________________

14) In what language do you prefer your health information? __________________________________

15) How often do you typically visit the Emergency Room in a year?

- ◯ 0
- ◯ 1
- ◯ 2
- ◯ 3
- ◯ 4 or more

16) Have you or anyone you've known had a bad experience in the Emergency Room?

- ◯ Yes
- ◯ No

Please Explain. __________________________________

Several of the following questions will ask you to rank your feelings or beliefs regarding this visit. (e.g. strongly agree, agree, disagree, or strongly disagree.)

17) I could have waited 24 hours to be seen by any doctor for this health issue.
- ◯ Strongly Agree
- ◯ Agree
- ◯ Disagree
- ◯ Strongly disagree

18) My health issue could have been effectively and safely treated by a primary care doctor in a clinic.
- ◯ Strongly Agree
- ◯ Agree
- ◯ Disagree
- ◯ Strongly disagree

19) In the past, the information and directions given to me by Emergency Room doctors have been clear.
- ◯ Strongly Agree
- ◯ Agree
- ◯ Disagree
- ◯ Strongly Disagree
- ◯ N/A - I have not interacted with Emergency Room doctors yet.

20) Has COVID-19 prevented you from visiting the Emergency Room in the past?
- ◯ Yes
- ◯ No

21) How often do you encounter an Emergency Room doctor who listens to you and treats you with dignity and respect?
- ◯ Very Often
- ◯ Often
- ◯ Rarely
- ◯ Never
- ◯ Not Applicable (never encountered an Emergency Room doctor)

22) Generally speaking, do you think doctors, nurses, hospitals, or insurance companies treat people differently based on their race or ethnic background?
- ◯ Yes
- ◯ No

Please Explain Your Answer Choice: __________________________________

23) Do you have a Primary Care Doctor (a doctor that you can see regularly and make an appointment with)?
- ◯ Yes
- ◯ No

24) If yes, have you seen your Primary Care Doctor in the last 12 months?
- ◯ Yes
- ◯ No

25) Have you seen your doctor in the past 12 months for similar symptoms that you're having today?
- ◯ Yes
- ◯ No

26) How often do you visit your primary care physician in a year?
- ◯ 0
- ◯ 1
- ◯ 2
- ◯ 3
- ◯ 4 or more

27) I trust my primary care doctor.

- ◯ Strongly Agree
- ◯ Agree
- ◯ Disagree
- ◯ Strongly Disagree

28) The information and directions given to me by my primary care physician are clear.

- ◯ Strongly Agree
- ◯ Agree
- ◯ Disagree
- ◯ Strongly Disagree

29) Have any of these reasons prevented you from going to see your Primary Care Provider (PCP)? Check all that apply.

- ☐ COVID-19
- ☐ Transportation
- ☐ Wait Time for visit
- ☐ Limited appointment availability
- ☐ Cost
- ☐ Poor relationship with PCP
- ☐ Job responsibilities/My doctor's office isn't open at a time I can visit
- ☐ Caring for loved one
- ☐ Other

If other, please explain why:

__________________________________
